# Modern Dental Remedies

**Published:** 1907-02-15

**Authors:** E. T. Loeffler


### MODERN DENTAL REMEDIES.

BY E. T. LOEFFLER, B.S., D.D.S.

Read before the Detroit Dental Society, October 9, 1906.

Omitting all introductory remarks and taking it for granted that there can be no ambiguity in the meaning of the word Dental, let me briefly call your attention to the various definitions that are given to the word remedy.

In the broadest sense, a remedy is anything that will counteract or remove an evil of any kind—reparation, relief. It is a thing that will change the abnormal to the normal.

In a medical sense: Webster defines remedy as anything which cures diseases. The Standard Dictionary gives it as; that which is used in any way for the cure or reliefs of bodily disease; a medicine; a remedial treatment. Dorland says; a remedy is anything that cures, palliates, or prevents disease. In Funk and Wagnalls we find the definition; to cure or heal as by medicinal treatment. Harris defines; it a medicine employed for the prevention, alleviation or cure of disease.

Long, in his text-book on Materia Medica and Therapeutics, gives a very comprehensive and in some respects an ideal definition. He states that; the term remedy is quite inclusive, meaning any agent whether a substance, force, or any influence whatever, employed in the treatment of disease. It may be a medicine or any external force or influence. It may be intended for internal administration, for external application, or for less direct mental influence. "Nature heals and we simply treat"—is a well established truth that is often forgotten, especially by the patient who not infrequently asks us to cure, whereas all that we can do is to treat. It may be rather amusing to note the wide range of meaning in the term modern. We certainly would be justified in calling any remedy modern that has been used within the last fifty years. Just as modern history dates back to the middle ages. Modern simply means pertaining to the present period, not to some ancient period. Recent would have been, perhaps, a better term. It is not quite so sharply distinguished from the past as new. The term novel as applied to a remedy, would be quite characteristic; novel, however, means something that is unprecedented in kind; while that which is new is just produced, but may be of a familiar or even an ancient sort, as a new preparation of an old remedy. This in brief explains how difficult it is to word the subject of a paper so as to give it the desired meaning.

In speaking of modern dental remedies then we shall feel obliged to use the term remedy in its restricted meaning, that is, a medicinal agent that should be found in every up-to-date dental cabinet, to be applied for the relief of dental diseases.

It may be rather surprising, but the number of absolutely useful remedies that have been added to the dental pharmacopea during the past few years has been few. The same is true also in general medicine. Of course, the usual number of organic and synthetic preparations have made their appearance, especially in Germany, but thus far none have met with special favor. Perhaps this is due to the fact that experimenters and reputable practitioners are rather more conservative about proclaiming the advantages of any new agent than they used to be.

In spite of the fact that there are those who think otherwise, Ethyl Chloride, is rapidly coming to the front as an introductory anaesthetic. Better appliances and more experience are largely responsible for this fact. Some go so far as to say that it is rapidly replacing the more cumbrous nitrous oxide apparatus, and is, according to some authorities, quite as safe.

Scopolamin-morphine, as a general anaesthetic, has been disappointing, in fact the great claim made for it has, in a measure, fallen to the ground. Morphine and atropine answer the purpose quite as well. But as one writer puts it—"Like the Greeks we are always running after the old gods with new names."

It may seem out of place from the dentists' view point but it may be interesting to state that inunctions in the treatment of syphilis is almost a thing of the past.

Of the comparatively few cocaine substitutes that are now on market and awaiting further trials, none have received complete recognition. Competent authorities claim that their seeming good qualities are more than counterbalanced by their disadvantages.

As a general stimulant and nerve tonic, strychnine, either hypodermically or per stomach, is still considered as one of the very best.

During the past year, especially, there has been a marked increase in the tendency to secure medicinal agents from animals rather than from plants. It may be particularly interesting to the dental profession to know that our knowledge concerning the therapeutic value of the suprarenal glands has not increased. On the other hand it may be well to note that sloughing and various other undesirable complications have resulted from its subcutaneous injections. For example, the frequent use of its active principle, adrenalin chloride, has unquestionably a tendency to produce a disintigration in the walls of the arteries.

In regard to the X-Ray or Roentgen-Ray therapy, there has not been the usual amount of enthusiasm. There has, if anything, been a restriction in its field of usefulness.

Most of you, perhaps, understand that a streptococcus infection in the oral cavity is rather a serious condition. The serum, known as antistreptococcus has, for some time been considered in the light of an experiment. However, now we have every reason to believe that its use is followed by marked beneficial results. It seems to act much in the same manner as the well-known antitetanic serum, and in suspicious cases even better as a prophylactic than as a curative agent.

In the past year a number of new pharmacopias have appeared in Europe as well as in this country. As a result this has not created any particular disturbance, because previous issues are consulted, except by those who are particularly interested in new research work. In the new issue of the U. S. Pharmacopea the profession has become particularly interested. It seems that a number of agents which have heretofore been considered as extremely useful have been omitted, while many of less importance or not in general use have been retained.

By all means the greatest advance that has beeen made in therapeutics from a scientific viewpoint is the stand that has been taken against the so called nostrum or secret preparations. This has been largely the work of medical journals and other periodicals devoted to the interests of the profession. A committee on pharmacy and chemistry has been established by the American Medical Association, which investigates all new remedies and reports each week its findings in the Journal of the American Medical Association.

Why is it that proprietary remedies are so much more popular than others? Simply because they are more palatable and agreeable to the taste, and as a rule produce less disturbances of the digestive tract. By consulting our pharmacopeia, or National Formulary, or, perhaps with the assistance of our druggists we certainly might be able to have put up, remedies that are just as pleasant as these so-called proprietaries. Of course, in all this the dentist is not so keenly interested as the general practitioner. In a number of cities active steps have been taken in this direction. Druggists take more pride in putting up their prescriptions, and such a movement is bound to result in much good.

The following is a list of remedies used by the dental students in the college; 1. Carbolic Acid; 2. Oil of Cloves; 3. Oil of Eucalyptus; 4. Black's, 1,2,3; 5. Aconite and Iodine; 6. Iodized Phenol; 7. Phenol-sodique; 8. Camphophenique; 9. Tincture of Iodine; 10. Aromatic Spirits of Ammonia; 11. Aromatic Sulphuric Acid; 12. Arsenious Acid 13. Formalin 1 part and Tri-cresol, 3 parts; 14. Chlora Percha; 15. Tri-chlor-acetic Acid; 16. Glycerite of Tannin; 17. Bicarbonate of Soda; 18. Sandarac Varnish; 19. Peroxide of Hydrogen; 20. Dialized Iron; 21. Cocaine Crystals; 22. Nitrate of Silver; 23. Ammonia. There are a few other drugs as adrenalin chloride, pyrozone, dentalone, stypticin thymophene and thymocamphene, that we use in our clinics, but do not ask the students to furnish them on account of the added expense and the instability of a part of them.

Many of these agents, as carbolic acid, for example, are really old remedies, but used in a new way. There is absolutely no drug that possesses the wide range of usefulness that this one does. As a caustic, antiseptic, germicide and local anaesthetic it cannot be replaced by any. When used hot or in combination with cocaine or as camphophenique it serves as one of the best obtundents of sensitive dentine.

Oil of cloves still has the reputation of being one of the best drugs to be used in allaying the pain due to an irritated or inflamed pulp.

Oil of eucalyptus, in the filling of root canals with gutta-percha has great value on account of its solvent and non-staining powers. But for the reason that it often sets up a slight percementitis it has lost much of the reputation it formerly had.

Black's 1-2-3, 1 part of cassia, 2 parts carbolic acid, and 3 parts oil of wintergreen, as an antiseptic and root canal dressing, has won a world-wide reputation. It possesses excellent disinfectant qualities not represented by either drug alone.

Tincture of aconite and tincture of iodine equal parts (sometimes an equal part of chloroform is added) is almost universally used for painting the gums in acute pericementitis. The sedative action of the aconite and chloroform, when used, and the stimulating effect upon the lymphatics of the iodine makes this, an ideal remedy for such purposes. In the treatment of abscesses with fistulour openings there is no medicament used by the dental profession today that can compare with iodized phenol. It might be considered a specific in the true sense of the word for all such pathological conditions. It consists of phenol and tincture of iodine in varying proportions. When used in equal parts it is sometimes used as an efficient counterirritant.

Phenol-sodique, although a proprietory preparation corresponds very closely to the combination known as, liquid sodii carbolatis, which contains the following; phenol, 50 parts; sodium hydroxide, 3.5 parts and water 46.5 parts. It is a rather unstable compound and should be freshly made at intervals. It has its great field of usefulness in combination with pulverized pumice stone in the work of oral prophylaxis. Its alkaline reaction together with its stimulation and antiseptic qualities produce the good results that are witnessed almost daily by those who are interested or engage in this line of work.

Camphorated phenol, or campho-phenique, consisting of equal parts of camphor and phenol. It is less soluble than phenol, and is often used instead of the latter where a stimulating rather than a depressing action is desired.

Tincture of iodine is a typical example of a counterirritant. It is said that a solution of tincture of iodine, one dram to the pint of water, giving it the color of good brandy, is a most excellent mouth wash after extractions. This comparatively new value of iodine has, I am afraid not been fully appreciated.

Aromatic spirits of ammonia as a ready and handy cardiac and respiratory restorative cannot easily be replaced by any other remedy of the same class.

Sulphuric acid when used in connection with bicarbonate of soda as in Dr. Callahan's method for cleansing putrescent root canals or for other similar purposes is lauded the world over; while bicarbonate of soda, used in place of phenol-sodique in oral prophylaxis, especially when the necks of teeth are very sensitive, gives results that are very gratifying.

There are some who advocate discarding the use of arsenious acid as a nerve paste, especially since the advent of cocaine and the high pressure anaesthetic methods. However, we think it still holds a valued place in certain cases.

Formolin 1 part and trikresol 3 parts in the treatment of putrescent pulps, blind abscesses, and in chronic pericementitis give excellent results. For some time we used formolin and trikresol in equal parts, but found the formolin too irritating. It is certainly worth while for those interested to read the articles on this preparation written by Dr. Buckley, of Chicago.

Chlora-percha has not been displaced by any root canal filling. Its many good features are familiar to all. Eucalyptol in the proportion of from 5—10 drops to the ounce added to chlor-percha is considered to be a better preparation than the chlora-percha alone.

Tri-chlor-acetic acid (Merck & Co.) in full strength is without a peer as a caustic for removing superfluous gum tissue found so frequently in proximal cavities. In a 10% solution, reliable authorities claim that it has no equal in the treatment of pyorrhoea alveolaris.

Glycerite of tannin serves its best purpose as a final dressing to irritated gum tissues especially after an operation where the rubber dam clamps and separators have been used. It acts in the capacity of an antiseptic and protective.

Sandarac Varnish is still used quite largely with cotton as a temporary stopping, also for protecting cement fillings and inlays.

Hydrogen peroxide, or Dioxygen, is used a great deal by our students as a detergent disinfectant in treating putrescent pulps or in the removal of the same generally. For removing stains it is one of the best agents to be used in combination with pulverized pumice stone.

Of the dialized iron the best preparation to be used is the Parke, Davis & Co. in the form of a thick syrup, and this only in case of arsenical poisoning.

The many uses of cocaine hydrochlorate are so well understood that it scarcely needs any mention here. None of the various substitutes have so far been able to displace it.

Nitrate of silver has its special field of usefulness in the treatment of children's teeth to temporarily arrest decay.

Aqua ammonia is principally used for removing stains as from iodine and also in the process of bleaching teeth, preceding the use of pyrozone, which without doubt is the ideal agent in this kind of work. It is also a valuable resuscitant.

The action and worth of adrenalin chloride has been so fully explained in the various journals that it seems unnecessary to make any special mention of it at this time. It is a valuable dental styptic, and is much used in connection with cocaine in painless pulp extermination.

In the field of organic chemistry there has originated some of the most highly prized drugs. It is said that the chemists are being overtaxed in the search after new medicine compounds. Many of the synthetic compounds have been disappointing, they seem to be all right from a chemical point of view, but when physiological tests are made they prove to be almost worthless.

We are all aware what a tendency there is to offer to the profession new remedies. Some of these are not new at all, but simple old compounds with some slight modifications. Acetanilid and the different preparations containing it is a striking illustration of this practice, and have a useful anodyne place as dental remedies. I desire to make an earnest appeal to the profession to refrain, or absolutely refuse to use or indorse any preparation whose formula is not given, especially as we have at our disposal many known remedies which serve us just as well, if not better. There is no good reason why any dentist should buy or use any of the so-called abscess cures that are on the market.

